# Zero-dose children and the extended immunisation cascade: Understanding the path to full immunisation with six childhood vaccines in 43 countries

**DOI:** 10.7189/jogh.14.04199

**Published:** 2024-09-27

**Authors:** Bianca O Cata-Preta, Larissa Adna Neves Silva, Francine Santos Costa, Thiago Melo Santos, Tewodaj Mengistu, Daniel R Hogan, Cesar Gomes Victora, Aluisio JD Barros

**Affiliations:** 1 Department of Public Health, Federal University of Parana, Curitiba, Parana, Brazil; 2 International Center for Equity in Health, Federal University of Pelotas, Pelotas, Rio Grande do Sul, Brazil; 3 Gender and Women’s Health Unit, Nossal Institute for Global Health, School of Population and Global Health, University of Melbourne, Melbourne, Australia; 4 Gavi, the Vaccine Alliance, Global Health Campus, Geneva, Switzerland

## Abstract

**Background:**

As part of the Immunisation Agenda 2030, the World Health Organization set a goal to reduce the number of children who did not receive any routine vaccine by 50% by 2030. We aimed to describe the patterns of vaccines received for children with zero, one, and up to full vaccination, while considering newly deployed vaccines (pneumococcal conjugate vaccine (PCV) and rotavirus (ROTA) vaccine) alongside longstanding ones such as the Bacille Calmete-Guérin (BCG), diphtheria, tetanus, and pertussis (DPT), and poliomyelitis vaccines, and measles-containing vaccines (MCVs).

**Methods:**

We used data from national household surveys (Demographic and Health Surveys and Multiple Indicator Cluster Surveys) carried out in 43 low- and middle-income countries since 2014. We calculated the immunisation cascade as a score ranging from zero to six, considering BCG, polio, DPT, and ROTA vaccines, and the MCV and PCV. We also described the most prevalent combination of vaccines. The analyses were pooled across countries and stratified by household wealth quintiles.

**Results:**

In the pooled analyses with all countries combined, 9.0% of children failed to receive any vaccines, 58.6% received at least one dose of each of the six vaccines, and 47.2% were fully vaccinated with all doses. Among the few children receiving 1–5 vaccines, the most frequent were BCG vaccines, polio vaccines, DPT vaccines, PCV, ROTA vaccines, and MCV.

**Conclusions:**

Targeting children with their initial vaccine is crucial, as those who receive a first vaccine are more likely to undergo subsequent vaccinations. Finding zero-dose children and starting their immunisation is essential to leaving no one behind during the era of Sustainable Development Goals.

Immunisation has played a key role globally in improving child health, particularly in low- and middle-income countries (LMICs), where coverage of new vaccines has increased substantially since 2000. However, despite this progress, routine immunisation services have faced challenges in extending their reach to all children, resulting in coverage levels below the World Health Organization's (WHO) Immunisation Agenda 2030 (IA2030) global target of 90% coverage by 2030 for vaccines across the life course, including three doses of the diphtheria, pertussis, and tetanus (DPT) vaccine and three doses of the pneumococcal conjugate vaccine (PCV) [1–3]. This is all the more concerning, as immunisation is one of the most cost-effective health interventions for enhancing global child survival rates [4]. A critical aspect of this challenge is the existence of ‘zero-dose’ children – a term referring to children who failed to receive a single dose of a recommended vaccine. As part of the IA2030, the WHO set a goal of achieving a 50% reduction in the number of zero-dose children by 2030, relative to levels in 2019 [5].

In a previous publication, we introduced a novel framework – the ‘immunisation cascade’ – to understand the pathway from zero-dose to full immunisation coverage in the population. We described which vaccines were most likely to be received by children who received one, two, three, or four vaccines, and the correlations between them [6]. In doing so, we focussed on four types of well-established infant vaccines – the Bacille Calmete-Guérin (BCG) vaccine, the poliomyelitis vaccine, DPT-containing vaccines, and measles-containing vaccines (MCVs). Using household survey data from 92 LMICs, we estimated the percentage of zero-dose and fully immunised children, as well as the percentages of children who were partially vaccinated along the immunisation cascade. The results showed that children with one vaccine are most likely to have received a BCG vaccine, and that BCG and polio vaccines are the most common combination in children with two vaccines. The estimates suggested that 7.7% of all children were zero-dose; 3.3%, 3.4%, and 14.7% received at least one dose of one, two, or three vaccines, respectively; 70.9% received at least one dose of all four vaccines; and 59.9% were fully vaccinated, having received all prescribed doses of the four vaccines.

In the last decade, many countries introduced additional vaccines in their immunisation schedules, particularly PCVs and ROTA vaccines. Here we expand the immunisation cascade analyses to incorporate these newer vaccines, in addition to the four described above. Specifically, we describe the characteristics of the seven-level cascade among LMICs with different levels of economic development, and we also address within-country socioeconomic inequalities. These analyses highlight equity gaps in immunisation and potential opportunities to ensure zero-dose and under-vaccinated children become fully vaccinated.

## METHODS

We used data from Demographic and Health Surveys (DHS) and Multiple Indicator Cluster Surveys (MICS) conducted from 2014 to 2021. The DHS and MICS are population-based surveys with high response rates, rigorous interviewer training, and standardised sampling design and data collection procedures across countries [7,8]. We extracted data from the coverage indicator database of the International Center for Equity in Health at the Federal University of Pelotas, which includes all publicly available surveys carried out since the 1990s. We used this database to identify all surveys from LMICs with information on the six vaccines under study. No surveys prior to 2013 met our inclusion criteria.

Coverage estimates were based on children aged 12 to 23 months. In the case of Peru in 2022, the age range was 18–29 months, considering the age of measles vaccination in the country.

Information on vaccine status in the DHS and MICS was collected from vaccination cards whenever available; otherwise, they were obtained through the primary caregiver's report. We considered children without any information on vaccination as unvaccinated, as recommended by the WHO. We also calculated the immunisation information based upon caregiver’s recall only; on average, 18% of immunisation information was based upon caregiver’s recall (Table S6 in the **Online Supplementary Document**).

The analyses considered six vaccines: the BCG, DPT, polio (either oral or injectable) and ROTA vaccines, the MCV, and the PCV. We examined the number of children who received the three recommended doses for the polio and DPT vaccine, and the PCV. Full ROTA immunisation was defined as two or three doses, according to the product used in each country. A child was considered fully immunised if they had received a full course of all vaccines (one dose of the BCG vaccine and the MCV each, three doses of DPT-containing vaccines, the polio vaccine, and PCV each, and two or three doses of the ROTA vaccine).

We expressed the immunisation cascade as a score ranging from zero to six, with each type of vaccine contributing one point to the cascade, regardless of the number of doses. For example, a child was assigned a score of 1 if they had received only one of the vaccines, irrespective of the number of doses; therefore, if they only received polio vaccine either one, two or three doses, they were assigned as level one of the cascade. We then estimated percentages for each of the seven cascade levels, indicating the proportion of children with the corresponding number of vaccines received. For each cascade level, we also calculated percentages of all possible combinations of different vaccines.

Co-coverage with combinations of each pair of vaccines was calculated as the conditional probability of receiving one vaccine given that the other was received. For instance, we presented the proportion of children receiving the polio vaccine, given they received the BCG vaccine. We did so for all vaccine two-by-two combinations. Similarly, we presented the proportions of children who received a given vaccine conditional to not having received another one.

We further derived conditional probabilities for the first and third doses of the DPT vaccine, polio vaccine, and PCV. For the ROTA vaccine, the first and the second or third doses were considered based on the country's vaccine schedule. We also calculated conditional probabilities for receiving a vaccine when another vaccine was not received.

We also explored within-country inequalities based on household wealth quintiles, provided in the DHS and MICS data sets. The household wealth index was derived from household assets, building characteristics, availability of electricity, water supply, sanitary facilities, and other wealth-related variables using principal component analyses. Since relevant assets can differ between urban and rural households, principal component analyses were performed separately for each area and then combined into a single score using a scaling procedure to ensure comparability between areas. The households were then sorted by the wealth index and divided into five groups of equal population size (quintiles). The first quintile includes the households with the poorest 20% of the sample, up until the fifth quintile with the 20% wealthiest [9].

We performed sensitivity analyses comparing the 15 countries where PCV or ROTA vaccination had been introduced up to three years before the survey (where coverage may still be scaling up) to the remaining 28 countries. The introduction dates for the PCV and the ROTA vaccine were gathered at the VIEW-hub platform [10]. (**Online Supplementary Document**).

We performed statistical analyses in Stata, version 18.0 (StataCorp LLC, College Station, TX: StataCorp) and R, version 4.1.0. (R Core Team, Vienna, Austria), accounting for the multi-stage survey design and incorporating sampling weights and strata. Pooled results for all countries were weighted proportionally to the national population of children aged 12 to 23 months in 2019 (the median year of the surveys) [11].

## RESULTS

Forty-three countries (16 (37%) low-income, 19 (44%) lower-income), 8 (19%) upper-middle income) had surveys (18 MICS and 25 DHS or similar surveys) containing the required information on the six vaccines (Table S1 in the **Online Supplementary Document**). Regarding the years of introduction of the PCV and the ROTA vaccine, they were introduced in the same year in nine countries; the PCV was introduced first in 27 countries, while the ROTA vaccine was introduced before the PCV in the remaining seven countries (Table S1 in the **Online Supplementary Document**).

**Figure 1:**
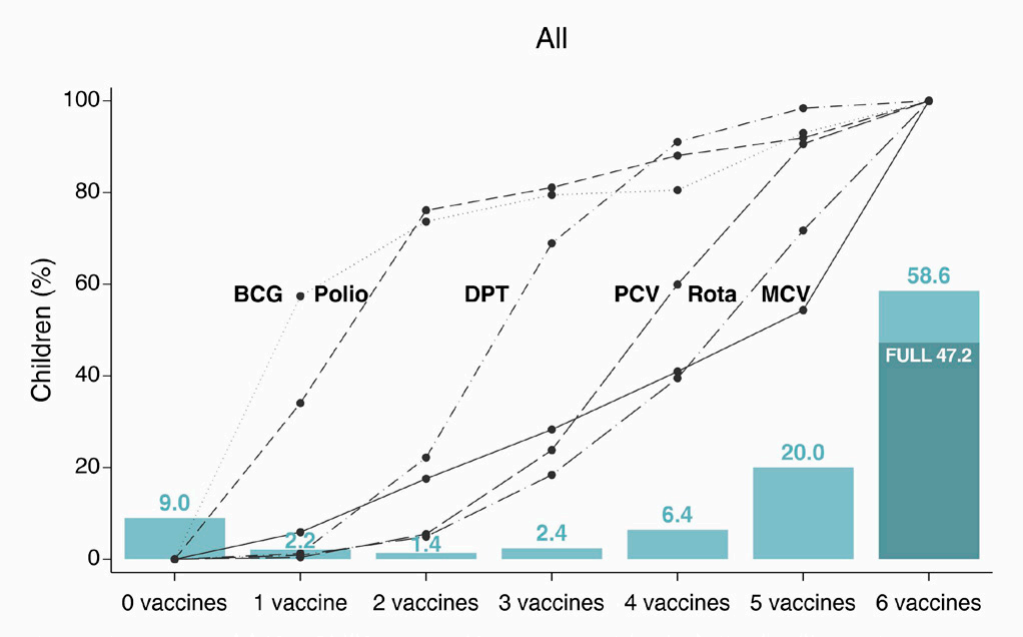
Percent of children in each level of the immunisation cascade. The bars represent the percentage of children in each level of the immunisation cascade, while the lines represent the percentage of children who received each vaccine at a given level of the cascade. The last bar shows the proportion of fully vaccinated children in a darker shade, while the entire bar represents the proportion of children who received the six vaccines but not necessarily all recommended doses.

**Figure 1** shows the combined extended immunisation cascade aggregated across 43 countries. For children at level 1, 57.4% received the BCG vaccine, another 34.1% received one dose of the polio vaccine, and fewer than 6% received any other type vaccine (Tables S2–3 in the **Online Supplementary Document**).

Across all countries with data, 9.0% of the children had received no vaccination. The proportions of all children with one, two, or three vaccines were below 3%, while 6.4% had four and 20.0% had five vaccines. Just over half (58.6%) of all children had received the six vaccines, and 47.2% were fully immunised. The main difference between levels 5 and 6 is low coverage with the MCV, which seems to be the main obstacle to receiving all vaccines.

In the 28 countries with earlier (four years or more) introduction of the PCV and the ROTA vaccine, full immunisation coverage was 1.8 times higher than in the 15 countries with more recent introduction, where there were virtually the same percentage of children in level 5 and 6 (Figures S1 and S2 in the **Online Supplementary Document**). Additionally, the ordering of vaccines at levels 3 to 5 varies: in countries with earlier implementation, the MCV was the least common vaccine at these levels, whereas ROTA vaccine showed the lowest coverage in countries with recent introduction.

BCG was the most frequently received vaccine by children who received a single vaccine, followed by polio (**Figure 1**, **Table 1**). After BCG and polio vaccines, the following levels of the cascade included the DPT vaccine, the PCV, the MCV, and the ROTA vaccine, in this order (Table S4 in the **Online Supplementary Document**).

**Table 1:** The most frequent combinations of vaccines in each cascade level*

Cascade of vaccines	Vaccines combinations	Percentage of all children	n
0	None	9.0	6971
1	BCG	1.2	1092
2	BCG + POLIO	0.8	652
3	BCG + POLIO + DPT	1.0	836
4	BCG + POLIO + DPT + PCV	1.7	1211
5	BCG + POLIO + DPT + PCV + MCV	9.1	7630
6	BCG + POLIO + DPT + PCV + MCV + ROTA	58.6	48 451

*The percentages of children do not add up to 100% because only the most common combinations of vaccines at each level are shown. For the full list of combinations, see Table S4 in the **Online Supplementary Document**.

BCG – Bacille Calmete-Guérin, DPT – diphtheria, pertussis, and tetanus, MCV – measles-containing vaccine, PCV – pneumococcal conjugate vaccine, POLIO – poliomyelitis vaccine, ROTA – rotavirus vaccine

**Figure 2:**
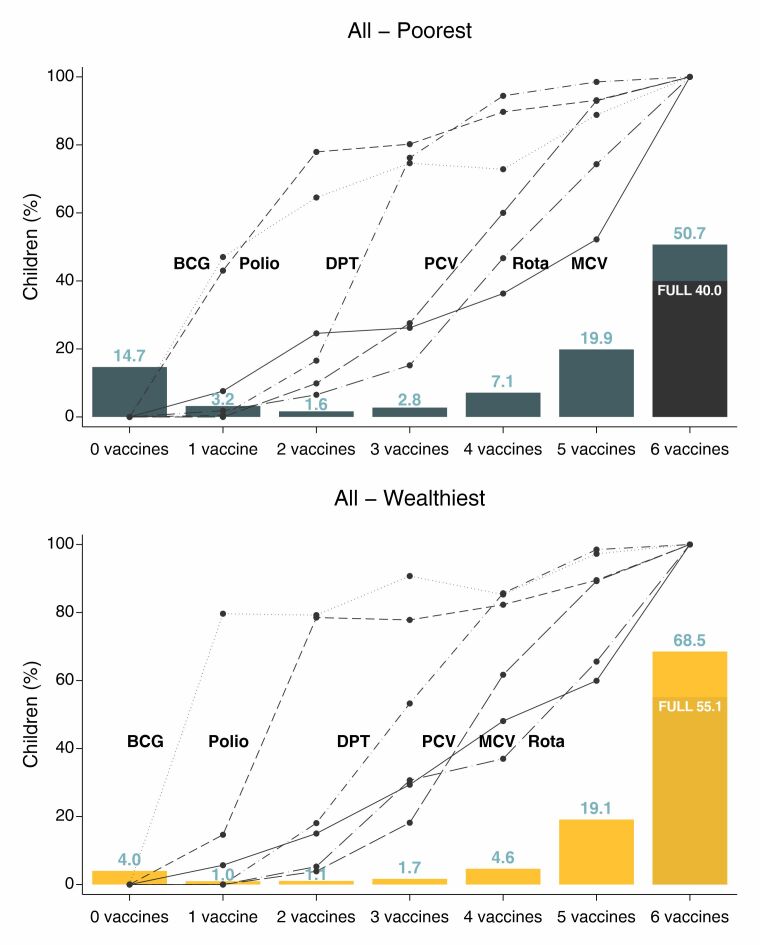
Percent of children in each level of the immunisation cascade. The bars represent the percentage of children in each level of the immunisation cascade in the poorest and wealthiest quintiles, while the lines represent the percentage of children who received each vaccine at a given cascade level.

While zero-dose prevalence and full immunisation coverage show significant differences among the poorest and wealthiest children, our analysis reveals similarities in their vaccination cascade patterns (**Figure 2**; Table S5 in the **Online Supplementary Document**.). The main difference is that – for poor children – the polio vaccine tends to have higher coverage than the BCG vaccine at each cascade level, whereas the reverse is true for children from wealthy families. The ordering of the remaining vaccines is consistent in both groups: the DPT vaccine, the PCV, the ROTA vaccine, and the MCV, although the ROTA vaccine and MCV were very close in the wealthiest group. For both groups of children, zero-dose prevalence is more common than receiving one, two, or three vaccines, and level six concentrates the largest proportions of children. Lastly, about 40% of the poorest and 55% of the wealthiest children in level six are fully immunised.

**Figure 3:**
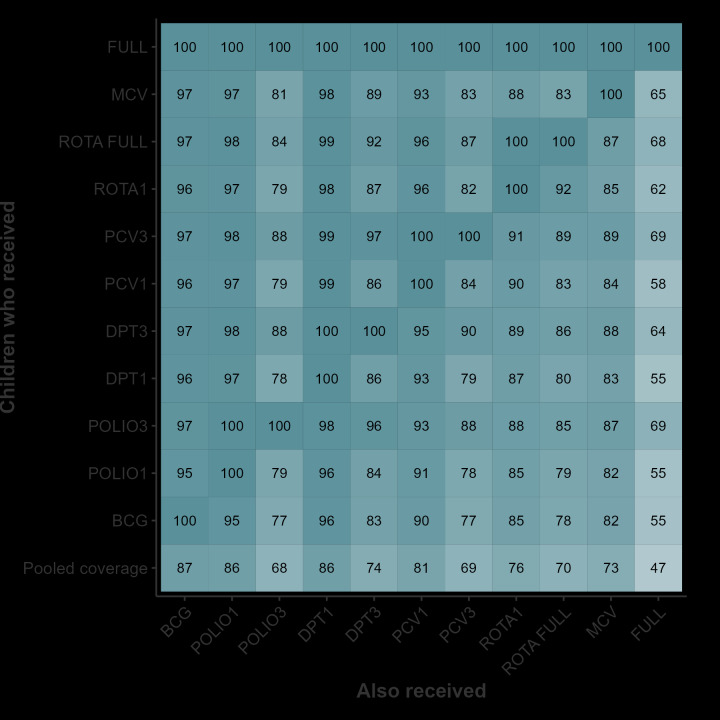
Conditional probability of receiving a given vaccine according to having received another vaccine. Pooled analyses of 43 countries. The figure shows the probability of receiving a given vaccine among children who had received another vaccine. In addition to pairs of different vaccines, the plot also shows children who received the third dose of polio, DPT, and PCV, and the second or third dose of ROTA vaccine (rota full), given they had received the first dose of the same vaccine. Darker colour shades in cells represent higher conditional probabilities.

**Figure 3** shows the conditional probabilities for each pair of vaccines, that is, the probability of receiving a given vaccine among children who had received another vaccine. In addition to pairs of different vaccines, the plot also shows children who received the third dose of polio, DPT, and PCV, and the second or third dose of ROTA vaccine (rota full), given they had received the first dose of the same vaccine. Darker colour shades in cells represent higher conditional probabilities. For example, the probability of receiving three doses of the polio vaccine among children who received the BCG vaccine is 77%.

Looking at the probability of being fully immunised among children who received a given vaccine, we found that it ranged from 55% for children who had received the BCG vaccine, one dose of the DPT vaccine, or one dose of the polio vaccine to 69% for children who had received three doses of the polio vaccine or three doses of the PCV immunisation.

Children who received one dose of any vaccine were very likely – 90% or higher – to receive at least one dose of the remaining vaccines, for example, 93% of children with one dose of the DPT vaccine also received one dose of the PCV. This was less marked for three doses of the polio vaccine (received by 77–88% of children who had received one dose of the remaining vaccines) and three doses of the PCV (77–90%).

As can be seen from **Figure 3**, 86% of children received the third dose of the DPT vaccine among those who had received the first, corresponding to a drop-out rate of 14%. For the PCV and the ROTA vaccine, drop-out rates were 14% and 8%, respectively. The highest drop-out rate of 21% was observed for polio vaccine. Looking at vaccines that should be delivered on the same occasion, such as the DPT vaccine and the PCV, we found that 90% of children who received three doses of the DPT vaccine also received three doses of the PCV.

**Figure 4:**
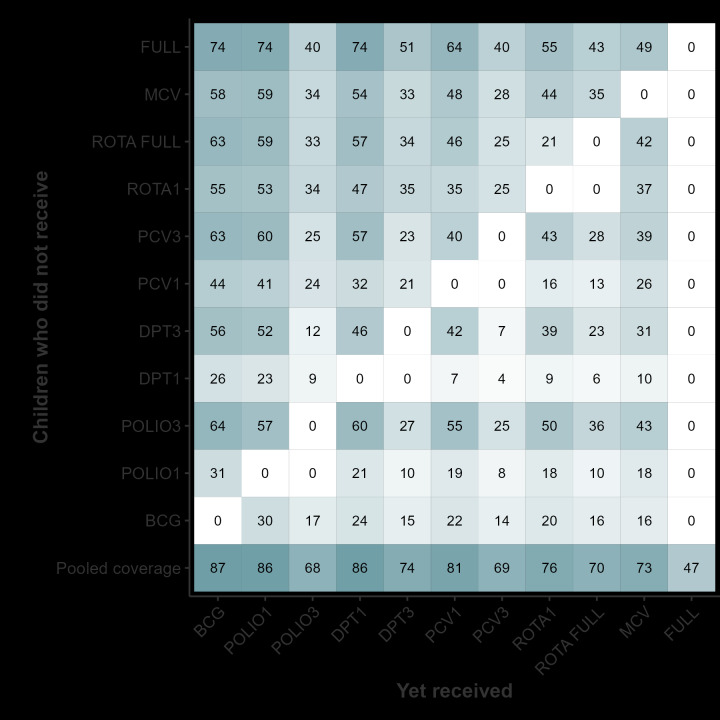
Conditional probability of receiving a given vaccine among children who failed to receive another vaccine. Pooled analyses of 43 countries.

**Figure 4** shows the probability of receiving a given vaccine among children who failed to receive another vaccine. Only 7% and 4% of children who were not vaccinated with one dose of the DPT vaccine received the first and third doses of PCV, respectively. In contrast, 48% and 28% of children not vaccinated with MCV received the first and third doses of PCV. One dose of the DPT vaccine was also a stronger predictor than the MCV of receiving the ROTA vaccine: only 9% of children who failed to receive one dose of the DPT vaccine were vaccinated against ROTA (first dose), compared to 44% of those did not receive the MCV.

The lighter colour rows in **Figure 4** for BCG, polio1, and DPT1 indicate that few children who failed to receive these vaccines were immunised with other vaccines. The top row shows that - among children who were not fully immunised – 74% had received the BCG vaccine, but only 43% had received two or three doses of the ROTA vaccine.

## DISCUSSION

The immunisation cascade is a new approach to understanding the dynamics of immunisation coverage among children from LMICs. In an earlier analysis of four long-available vaccines, we had shown that 7.7% of children aged 12–23 months from 92 countries were in the zero-dose category. Few children had received only one or two types of vaccines, while 15% received three and 71% four vaccines, indicating a J-shaped distribution. This pattern was interpreted as suggesting that moving children out of the zero-dose status will likely lead them to receive several immunisations, thus contributing to universal coverage [6]. In those analyses, the J-shaped pattern was particularly pronounced among children from poor families in low- and lower-middle-income country groups, compared to children from wealthier countries and families.

This analysis incorporated two additional, more recently introduced vaccines against pneumococcus and ROTA. Due to less information being available in national surveys on the newer vaccines relative to the previous studies, we were able to analyse only 43 countries. They slightly differ from the previous study in terms of United Nations International Children's Emergency Fund (UNICEF) region and World Bank income classification country distribution. Specifically, they were predominantly from African regions, with West and Central Africa, and Eastern and Southern Africa representing 34.9% and 32.5% of the sample, respectively. In the broader comparison group of 92 countries, these regions accounted for 24% and 20%, respectively. Eastern Europe and Central Asia were significantly less represented in the expanded cascade group, making up only 2% of the countries, compared to 13% in the general group. In terms of World Bank classification, lower-middle income countries were represented by 44% in the Expanded cascade group and 44% in the general group.

The J-shaped distribution persisted in our new analysis, however, with 9.0% of zero-dose children, few children with one, two, or three vaccines, 6.4% with four, 20.0% with five, and 58.6% with all types. As in the earlier analyses, the pattern was more pronounced among poor children due to higher zero-dose prevalence. In terms of receipt of at least one dose of a vaccine, the BCG and polio vaccines were the first two antigens with the highest coverage in the cascade, followed by the DPT vaccine, the PCV, the ROTA vaccine, and the MCV. Measles vaccination, therefore, represents the main obstacle to achieving full immunisation status, likely due to its position in the vaccination schedule, as it is typically administered last, around 9 or 12 months of age (Table S1 in the **Online Supplementary Document**).

Our results on inequalities are consistent with the literature, with zero-dose prevalence being higher in low-income countries and in children from poor families within a given country [6,12]. Nevertheless, the sequential orders of vaccines in the cascades were similar for rich and poor children, with the BCG and polio vaccines at the beginning and with the MCV and the ROTA vaccine at the end of the sequence.

Our results on conditional probabilities included not only first doses but also third doses of the polio and DPT vaccines, and the PCV, and second or third doses of ROTA vaccines, depending on the national schedules. Children who received a first dose of any vaccine tended to have a 90% or higher probability of receiving the first dose of all other vaccines, except for the ROTA vaccine and, to a lesser extent, the MCV. For multi-dose vaccinations, drop-out rates between the first dose and last dose needed for full immunisation ranged from 8% for the ROTA vaccine to 21% for the polio vaccine, with the DPT vaccine and the PCV being within this range. The lower drop-out for the ROTA vaccine has to be interpreted with caution as in 34 of the 43 countries only two doses are recommended, compared to three doses for the other vaccines. The high drop-out rate may be partly attributed to the difficulty some caregivers have in accessing health services, as multiple contacts are needed to complete vaccination schedules [13]. Attending the recommended number of antenatal care visits presents a similar pattern, with fewer visits being much more common than all the prescribed visits [14]. Additionally, caregivers may forget they need to bring the child for more doses or mistakenly believe that their child is already fully vaccinated.

The analyses of 28 countries that introduced the PCV and the ROTA vaccine four or more years before the survey showed higher coverage with these vaccines than in the 15 countries with more recent introduction, as one would expect in light of the time required for scaling up. In addition, in 28 countries, coverage with the PCV and the ROTA vaccine was higher than that for the MCV, whereas in the remaining 15 countries, the ROTA vaccine showed the lowest coverage among all vaccines.

Our analyses have some limitations. We analysed surveys from a wide range of years (2014 to present), with a median date of 2019; therefore, our results may not represent the current vaccination scenario in a given country. For instance, it is possible that the updated vaccination coverage has improved in a given country. Compared to the earlier analysis of 92 countries, only 43 could be included due to a lack of survey data on coverage with newer vaccines, particularly ROTA. In addition, about one in four children did not have a vaccination card for inspection, and information on immunisation was based on the mother’s recall, in agreement with international data analysis recommendations [15]. Recall bias is potentiated by the fact that several different vaccines are currently administered in the first year, in contrast to earlier surveys when only a few vaccines were routinely applied. There is evidence suggesting that recall-based coverage estimates tend to be higher than those based on vaccine cards [16]. Therefore, the scenario in LMICs may be less favourable than presented in this study. However, less than one in five doses of vaccine were based on report. The WHO recognises that understanding the reliability of recall as a source for vaccine coverage should be a research priority [17]. We treated children with missing information on vaccination as unvaccinated, according to the standard approach for estimating vaccine coverage from surveys [15].

Consistently reaching children who have not received any vaccines and reaching communities that may have been overlooked by routine immunisation while ensuring these children are fully vaccinated stands as a central objective in the IA2030 [18].

## CONCLUSIONS

According to our results, ensuring children receive their first vaccine is crucial, as those who receive a first vaccine are much more likely to undergo subsequent vaccinations. In this sense, country-specific investigations are essential to understand which are the individual and contextual factors driving the lack of the first vaccine. It can be, for instance, a lack of access to vaccination services or a lack of trust in vaccines. Stockouts are sometimes also an important problem, discouraging caregivers as they fear wasting money and time with an unsuccessful visit to the health facility. Addressing such challenges is the way to minimise zero-dose and under-vaccination [14,18,19]. Moreover, the analysis underscores the significance of reaching children who have not received any vaccines, addressing obstacles to under-immunisation, and emphasising that such efforts are likely to enhance equity in immunisation coverage. This is particularly important as poor children are more prone to being without any vaccines and experience higher rates of dropping out of immunisation programs. Successfully addressing these challenges would contribute significantly to the objective of leaving no one behind in immunisation during the era of the Sustainable Development Goals.

## Additional material


Online Supplementary Document
