# Supplementary material for: Zero-dose children and the extended immunisation cascade: Understanding the path to full immunisation with six childhood vaccines in 43 countries
Source: J Glob Health. 2024 Sep 27;14:04199. doi: 10.7189/jogh.14.04199 (PMC11426930; doi:10.7189/jogh.14.04199)
Supplement: Online Supplementary Document [file jogh-14-04199-s001.pdf]

SUPPLEMENTARY MATERIALS

Table S1. List of 43 countries, showing type and year of survey, number of children studied and PCV and rotavirus vaccine introduction dates.

Country	Year	Survey	Number of chi-ldren	Year of vaccine introduction	
				PCV	Rota
Afghanistan	2022	MICS	6177	2013	2018
Burkina_Faso	2021	DHS	2313	2013	2013
Cameroon	2018	DHS	1824	2011	2014
Cote_dIvoire	2021	DHS	1920	2014	2017
Dominican_Republic	2019	MICS	1674	2013	2012
El_Salvador	2014	MICS	1504	2010	2006
Eswatini	2021	MICS	422	2014	2015
Ethiopia	2019	DHS	1008	2011	2013
Fiji	2021	MICS	417	2012	2012
Gambia	2019	DHS	1582	2009	2013
Ghana	2022	DHS	1973	2012	2012
Guyana	2019	MICS	565	2011	2010
Honduras	2019	MICS	1711	2011	2009
Kenya	2022	DHS	3679	2011	2014
Madagascar	2021	DHS	2345	2012	2014
Malawi	2019	MICS	3206	2011	2012
Mali	2018	DHS	1946	2011	2014
Mauritania	2019	DHS	2119	2013	2014
Niger	2021	DHS	1619	2014	2014
Peru	2022	DHS	4200	2009	2009
Rwanda	2019	DHS	1572	2009	2012
Senegal	2019	DHS	1183	2013	2014
Sierra_Leone	2019	DHS	1861	2011	2014
South_Africa	2016	DHS	670	2009	2009
Tanzania	2022	DHS	2143	2012	2013
Yemen	2022	MICS	3741	2011	2012
Zambia	2018	DHS	1928	2013	2013
Zimbabwe	2019	MICS	1153	2012	2014
Angola	2015	DHS	2845	2013	2014
Armenia	2015	DHS	345	2014	2012
Benin	2021	MICS	2539	2011	2019
Burundi	2016	DHS	2596	2011	2013
Guatemala	2014	DHS	2408	2012	2010
Guinea_Bissau	2018	MICS	1409	2015	2015
Iraq	2018	MICS	3205	2016	2012
Kiribati	2018	MICS	453	2013	2015
Lesotho	2018	MICS	667	2015	2017
Liberia	2019	DHS	1063	2014	2016
Nepal	2022	DHS	1001	2015	2020
Sao_Tome_and_Principe	2019	MICS	349	2012	2016
State_of_Palestine	2019	MICS	1330	2011	2016
Togo	2017	MICS	973	2014	2014
Uganda	2016	DHS	2922	2013	2018

Legen: PCV – pneumococcal vaccine. Rota – rotavirus vaccine.

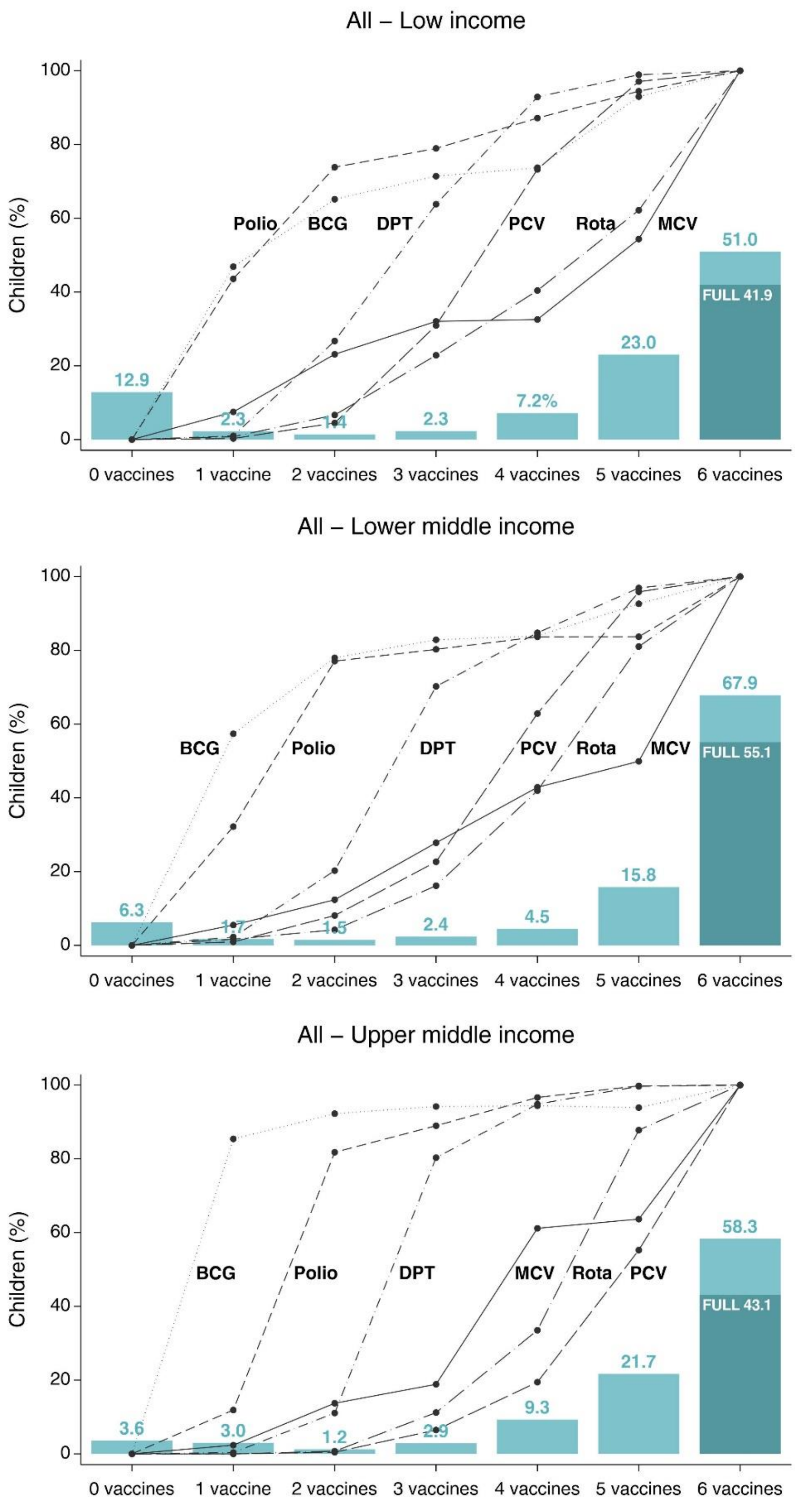

Figure S1. Immunization cascade in the 43 countries according to World Bank country income groups (2019 categorization)*.

(*) Because countries may change their classification over time, we opted to use the World Bank groupings for 2019, the median year of the surveys included in the analyses.

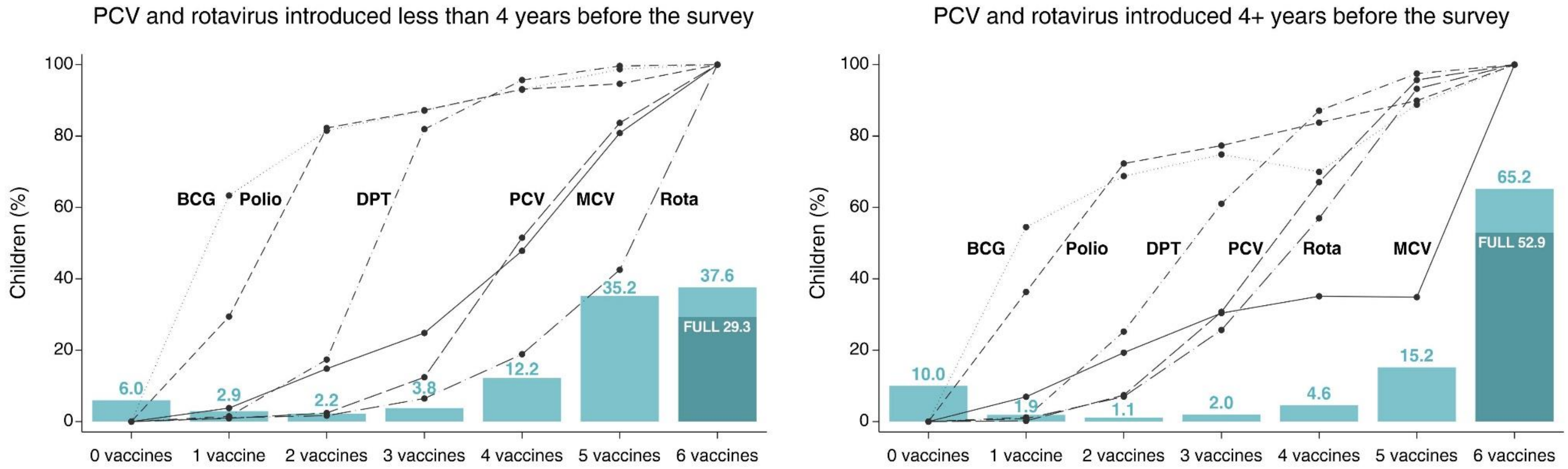

Figure S2. Immunization cascade in 15 countries where PCV and rotavirus vaccines were introduced less than 4 years before the survey, compared to 28 countries with vaccine introduction four or more 4 years before the survey.

Table S2 - Distribution of children by cascade level in the whole sample, poorest and wealthiest quintiles, with the 43 countries combined; 95% confidence intervals are shown for all estimates.

Cascade level	Sample of children	Percentage of children	Lower limit	Upper limit
0	Full sample	9.0	8.4	9.7
1	Full sample	2.2	1.9	2.4
2	Full sample	1.4	1.2	1.6
3	Full sample	2.4	2.2	2.7
4	Full sample	6.4	6.0	6.8
5	Full sample	20.0	19.4	20.7
6	Full sample	58.6	57.5	59.6
6	Full sample (fully immunized)	47.2	46.2	48.3
0	Poorest	14.7	0.1	0.2
1	Poorest	3.2	0.0	0.0
2	Poorest	1.6	0.0	0.0
3	Poorest	2.8	0.0	0.0
4	Poorest	7.1	0.1	0.1
5	Poorest	19.9	0.2	0.2
6	Poorest	50.7	0.5	0.5
6	Poorest (fully immunized)	40.0	0.4	0.4
0	Wealthiest	4.0	0.0	0.1
1	Wealthiest	1.0	0.0	0.0
2	Wealthiest	1.1	0.0	0.0
3	Wealthiest	1.7	0.0	0.0
4	Wealthiest	4.6	0.0	0.1
5	Wealthiest	19.1	0.2	0.2
6	Wealthiest	68.5	0.7	0.7
6	Wealthiest (fully immunized)	55.1	0.5	0.6

Table S3 – Vaccine coverage in each level of the immunization cascade in the pooled sample from 43 countries; 95% confidence intervals are shown for all estimates.

Cascade level	Vaccine	Coverage	Lower limit*	Upper limit*
0	BCG	0	0	0.01
0	DPT1	0	0	0.01
0	DPT3	0	0	0.01
0	MCV	0	0	0.01
0	PCV1	0	0	0.01
0	PCV3	0	0	0.01
0	Polio1	0	0	0.01
0	Polio3	0	0	0.01
0	ROTA1	0	0	0.01
0	ROTA FULL	0	0	0.01
1	BCG	57.4	51.8	62.8
1	DPT1	1.3	0.7	2.2
1	DPT3	0.3	0.1	0.7
1	MCV	5.9	4.5	7.7
1	PCV1	0.4	0.2	1.1
1	PCV3	0.1	0.0	0.3
1	Polio1	34.1	28.7	39.9
1	Polio3	13.4	10.3	17.2
1	ROTA1	0.9	0.5	1.8
1	ROTA FULL	0.5	0.2	1.3
2	BCG	73.7	67.7	78.9
2	DPT1	22.2	16.7	28.9
2	DPT3	9.1	4.7	17.1
2	MCV	17.6	14.3	21.4
2	PCV1	5.5	3.8	7.9
2	PCV3	1.0	0.3	3.4
2	Polio1	76.1	71.0	80.5
2	Polio3	24.5	19.5	30.4
2	ROTA1	4.9	3.5	7.0
2	ROTA FULL	2.2	1.4	3.4
3	BCG	79.5	74.2	83.9
3	DPT1	68.9	63.9	73.5
3	DPT3	28.2	23.8	33.0
3	MCV	28.3	24.2	32.7
3	PCV1	23.8	19.6	28.6
3	PCV3	7.4	4.7	11.4
3	Polio1	81.1	77.8	84.0
3	Polio3	38.0	32.8	43.5
3	ROTA1	18.4	14.3	23.4
3	ROTA FULL	11.4	7.7	16.5
4	BCG	80.5	77.1	83.5
4	DPT1	91.0	89.2	92.6

4	DPT3	52.9	49.5	56.3
4	MCV	40.9	37.6	44.4
4	PCV1	59.9	56.5	63.3
4	PCV3	29.3	26.1	32.7
4	Polio1	88.0	85.9	89.9
4	Polio3	47.7	44.7	50.6
4	ROTA1	39.5	36.2	42.9
4	ROTA FULL	25.5	22.4	28.9
5	BCG	93.0	92.0	93.9
5	DPT1	98.4	97.9	98.8
5	DPT3	75.8	74.1	77.5
5	MCV	54.3	52.5	56.2
5	PCV1	90.6	89.5	91.6
5	PCV3	63.5	61.6	65.4
5	Polio1	91.9	90.9	92.8
5	Polio3	68.1	66.5	69.6
5	ROTA1	71.8	70.4	73.1
5	ROTA FULL	59.5	57.9	61.0
6	BCG	100.0	99.9	100.0
6	DPT1	100.0	99.9	100.0
6	DPT3	93.5	92.2	94.5
6	MCV	100.0	99.9	100.0
6	PCV1	100.0	99.9	100.0
6	PCV3	91.7	90.7	92.7
6	Polio1	100.0	99.9	100.0
6	Polio3	85.8	84.7	86.7
6	ROTA1	100.0	99.9	100.0
6	ROTA FULL	95.3	94.8	95.8
6	VFULL	47.2	46.2	48.3

(*) 2 or 3 doses of rotavirus vaccine, depending on the national schedule.

* Wilson binomial confidence intervals were calculated for proportion of zero and 100.

Table S4 – Full list of combinations of vaccines in each cascade level.

Cascade	BCG	POLIO1	DPT1	PCV1	ROTA	MCV	% of all children	N
0	0	0	0	0	0	0	9.0%	6971
1	1	0	0	0	0	0	1.2%	1092
1	0	1	0	0	0	0	0.7%	612
1	0	0	1	0	0	0	0.0%	25
1	0	0	0	1	0	0	0.0%	7
1	0	0	0	0	1	0	0.0%	16
1	0	0	0	0	0	1	0.1%	128
2	1	1	0	0	0	0	0.8%	652
2	1	0	1	0	0	0	0.2%	119
2	1	0	0	1	0	0	0.0%	10
2	1	0	0	0	1	0	0.0%	10
2	1	0	0	0	0	1	0.1%	86
2	0	1	1	0	0	0	0.1%	68
2	0	1	0	1	0	0	0.0%	11
2	0	1	0	0	1	0	0.0%	31
2	0	1	0	0	0	1	0.2%	138
2	0	0	1	1	0	0	0.0%	22
2	0	0	1	0	1	0	0.0%	4
2	0	0	1	0	0	1	0.0%	9
2	0	0	0	1	1	0	0.0%	7
2	0	0	0	1	0	1	0.0%	3
2	0	0	0	0	1	1	0.0%	7
3	1	1	1	0	0	0	1.0%	836
3	1	1	0	1	0	0	0.1%	59
3	1	1	0	0	1	0	0.1%	83
3	1	1	0	0	0	1	0.3%	323
3	1	0	1	1	0	0	0.1%	90
3	1	0	1	0	1	0	0.0%	33
3	1	0	1	0	0	1	0.2%	140
3	1	0	0	1	1	0	0.1%	27
3	1	0	0	1	0	1	0.0%	15
3	1	0	0	0	1	1	0.0%	14
3	0	1	1	1	0	0	0.2%	131
3	0	1	1	0	1	0	0.0%	21
3	0	1	1	0	0	1	0.1%	62
3	0	1	0	1	1	0	0.0%	18
3	0	1	0	1	0	1	0.0%	10
3	0	1	0	0	1	1	0.1%	23
3	0	0	1	1	1	0	0.1%	36
3	0	0	1	1	0	1	0.0%	4
3	0	0	1	0	1	1	0.0%	3
3	0	0	0	1	1	1	0.0%	8
4	1	1	1	1	0	0	1.7%	1211
4	1	1	1	0	1	0	0.6%	474

Cascade	BCG	POLIO1	DPT1	PCV1	ROTA	MCV	% of all children	N
4	1	1	1	0	0	1	1.7%	1482
4	1	1	0	1	1	0	0.3%	116
4	1	1	0	1	0	1	0.1%	82
4	1	1	0	0	1	1	0.1%	130
4	1	0	1	1	1	0	0.3%	235
4	1	0	1	1	0	1	0.2%	148
4	1	0	1	0	1	1	0.1%	62
4	1	0	0	1	1	1	0.1%	51
4	0	1	1	1	1	0	0.9%	624
4	0	1	1	1	0	1	0.2%	140
4	0	1	1	0	1	1	0.1%	32
4	0	1	0	1	1	1	0.0%	12
4	0	0	1	1	1	1	0.1%	50
5	1	1	1	1	1	0	9.1%	7630
5	1	1	1	1	0	1	5.7%	3688
5	1	1	1	0	1	1	1.9%	1492
5	1	1	0	1	1	1	0.3%	219
5	1	0	1	1	1	1	1.6%	1200
5	0	1	1	1	1	1	1.4%	1067
6	1	1	1	1	1	1	58.6%	48451

Table S5 - Vaccine coverage in each level of the immunization cascade among children from the poorest and wealthiest quintiles in the pooled sample from 43 countries; 95% confidence intervals are shown for all estimates.

Cascade level	Wealth quintile	Vaccine	Coverage	Lower limit*	Upper limit*
0	Poorest	vbcg	0.0	0.0	0.15
0	Poorest	vdpt1	0.0	0.0	0.15
0	Poorest	vdpt3	0.0	0.0	0.15
0	Poorest	vmsl	0.0	0.0	0.15
0	Poorest	vpneumo1	0.0	0.0	0.15
0	Poorest	vpneumo3	0.0	0.0	0.15
0	Poorest	vpolio1	0.0	0.0	0.15
0	Poorest	vpolio3	0.0	0.0	0.15
0	Poorest	vrota1	0.0	0.0	0.15
0	Poorest	vrota_full	0.0	0.0	0.15
1	Poorest	vbcg	47.0	38.6	55.6
1	Poorest	vdpt1	0.5	0.1	1.6
1	Poorest	vdpt3	0.3	0.1	1.7
1	Poorest	vmsl	7.6	4.8	11.8
1	Poorest	vpneumo1	0.0	0.0	0.6
1	Poorest	vpneumo3	0.0	0.0	0.6
1	Poorest	vpolio1	43.0	34.2	52.4
1	Poorest	vpolio3	16.6	10.9	24.3
1	Poorest	vrota1	1.8	0.8	4.3
1	Poorest	vrota_full	1.0	0.3	3.4
2	Poorest	vbcg	64.5	56.1	72.2
2	Poorest	vdpt1	16.6	12.0	22.5
2	Poorest	vdpt3	3.9	2.2	6.9
2	Poorest	vmsl	24.6	17.7	33.1
2	Poorest	vpneumo1	9.9	6.1	15.5
2	Poorest	vpneumo3	0.4	0.1	2.9
2	Poorest	vpolio1	77.9	71.3	83.4
2	Poorest	vpolio3	22.7	17.5	28.8
2	Poorest	vrota1	6.5	3.6	11.5
2	Poorest	vrota_full	3.0	1.5	6.1
3	Poorest	vbcg	74.6	67.5	80.6
3	Poorest	vdpt1	76.2	70.3	81.2
3	Poorest	vdpt3	31.4	22.7	41.6
3	Poorest	vmsl	26.2	20.6	32.7
3	Poorest	vpneumo1	27.6	21.7	34.4
3	Poorest	vpneumo3	6.4	4.1	10.1
3	Poorest	vpolio1	80.2	74.3	85.0
3	Poorest	vpolio3	36.2	27.5	45.9
3	Poorest	vrota1	15.2	11.1	20.4
3	Poorest	vrota_full	7.0	4.6	10.7
4	Poorest	vbcg	72.9	66.7	78.2

4	Poorest	vdpt1	94.4	92.5	95.9
4	Poorest	vdpt3	52.3	46.8	57.8
4	Poorest	vmsl	36.3	31.6	41.3
4	Poorest	vpneumo1	60.0	54.3	65.5
4	Poorest	vpneumo3	27.9	22.8	33.7
4	Poorest	vpolio1	89.7	85.7	92.7
4	Poorest	vpolio3	46.4	40.9	51.9
4	Poorest	vrota1	46.7	41.1	52.4
4	Poorest	vrota_full	29.8	23.9	36.5
5	Poorest	vbcg	88.8	86.7	90.6
5	Poorest	vdpt1	98.5	97.8	99.0
5	Poorest	vdpt3	75.3	72.0	78.3
5	Poorest	vmsl	52.2	49.1	55.3
5	Poorest	vpneumo1	93.0	91.7	94.0
5	Poorest	vpneumo3	67.6	64.8	70.3
5	Poorest	vpolio1	93.1	91.8	94.3
5	Poorest	vpolio3	67.9	65.0	70.6
5	Poorest	vrota1	74.3	71.7	76.8
5	Poorest	vrota_full	63.1	60.1	66.0
6	Poorest	vfull_plus	40.0	38.5	41.4
6	Poorest	vbcg	100.0	99.9	100.0
6	Poorest	vdpt1	100.0	99.9	100.0
6	Poorest	vdpt3	93.0	91.8	94.0
6	Poorest	vmsl	100.0	99.9	100.0
6	Poorest	vpneumo1	100.0	99.9	100.0
6	Poorest	vpneumo3	90.6	88.9	92.1
6	Poorest	vpolio1	100.0	99.9	100.0
6	Poorest	vpolio3	84.4	82.5	86.1
6	Poorest	vrota1	100.0	99.9	100.0
6	Poorest	vrota_full	94.7	93.6	95.6
0	Wealthiest	vbcg	0.0	0.0	0.6
0	Wealthiest	vdpt1	0.0	0.0	0.6
0	Wealthiest	vdpt3	0.0	0.0	0.6
0	Wealthiest	vmsl	0.0	0.0	0.6
0	Wealthiest	vpneumo1	0.0	0.0	0.6
0	Wealthiest	vpneumo3	0.0	0.0	0.6
0	Wealthiest	vpolio1	0.0	0.0	0.6
0	Wealthiest	vpolio3	0.0	0.0	0.6
0	Wealthiest	vrota1	0.0	0.0	0.6
0	Wealthiest	vrota_full	0.0	0.0	0.6
1	Wealthiest	vbcg	79.6	68.7	87.4
1	Wealthiest	vdpt1	0.0	0.0	0.1
1	Wealthiest	vdpt3	0.0	0.0	0.1
1	Wealthiest	vmsl	5.7	2.4	13.0
1	Wealthiest	vpneumo1	0.0	0.0	2.2
1	Wealthiest	vpneumo3	0.0	0.0	2.2

1	Wealthiest	vpolio1	14.6	8.0	25.3
1	Wealthiest	vpolio3	11.2	5.3	22.2
1	Wealthiest	vrota1	0.0	0.0	2.2
1	Wealthiest	vrota_full	0.0	0.0	2.2
2	Wealthiest	vbcg	79.2	66.5	88.0
2	Wealthiest	vdpt1	18.1	10.0	30.4
2	Wealthiest	vdpt3	7.2	2.3	20.5
2	Wealthiest	vmsl	15.0	8.5	25.2
2	Wealthiest	vpneumo1	3.9	0.6	22.7
2	Wealthiest	vpneumo3	3.9	0.6	22.7
2	Wealthiest	vpolio1	78.5	65.2	87.7
2	Wealthiest	vpolio3	20.6	12.0	33.0
2	Wealthiest	vrota1	5.3	1.5	16.8
2	Wealthiest	vrota_full	0.9	0.1	5.9
3	Wealthiest	vbcg	90.7	83.1	95.1
3	Wealthiest	vdpt1	53.3	37.6	68.3
3	Wealthiest	vdpt3	26.9	17.5	39.1
3	Wealthiest	vmsl	29.4	19.6	41.5
3	Wealthiest	vpneumo1	18.2	11.1	28.3
3	Wealthiest	vpneumo3	6.8	3.2	13.9
3	Wealthiest	vpolio1	77.8	66.4	86.2
3	Wealthiest	vpolio3	47.8	33.0	63.0
3	Wealthiest	vrota1	30.7	15.5	51.6
3	Wealthiest	vrota_full	22.8	8.7	47.9
4	Wealthiest	vbcg	85.3	70.6	93.3
4	Wealthiest	vdpt1	85.7	79.2	90.4
4	Wealthiest	vdpt3	57.6	48.7	66.1
4	Wealthiest	vmsl	48.1	39.2	57.1
4	Wealthiest	vpneumo1	61.7	53.9	68.9
4	Wealthiest	vpneumo3	36.4	27.3	46.6
4	Wealthiest	vpolio1	82.3	76.4	87.0
4	Wealthiest	vpolio3	52.3	43.6	60.7
4	Wealthiest	vrota1	37.0	27.5	47.6
4	Wealthiest	vrota_full	29.9	20.3	41.6
5	Wealthiest	vbcg	97.3	96.1	98.1
5	Wealthiest	vdpt1	98.5	97.3	99.2
5	Wealthiest	vdpt3	79.4	76.2	82.3
5	Wealthiest	vmsl	59.9	54.0	65.6
5	Wealthiest	vpneumo1	89.2	86.9	91.2
5	Wealthiest	vpneumo3	64.6	60.0	68.9
5	Wealthiest	vpolio1	89.5	87.0	91.6
5	Wealthiest	vpolio3	68.4	64.4	72.2
5	Wealthiest	vrota1	65.6	60.7	70.2
5	Wealthiest	vrota_full	54.3	49.1	59.5
6	Wealthiest	vfull_plus	55.1	52.1	58.1
6	Wealthiest	vbcg	100.0	99.9	100.0

6	Wealthiest	vdpt1	100.0	99.9	100.0
6	Wealthiest	vdpt3	93.4	86.6	96.9
6	Wealthiest	vmsl	100.0	99.9	100.0
6	Wealthiest	vpneumo1	100.0	99.9	100.0
6	Wealthiest	vpneumo3	92.8	88.3	95.7
6	Wealthiest	vpolio1	100.0	99.9	100.0
6	Wealthiest	vpolio3	85.3	81.6	88.3
6	Wealthiest	vrota1	100.0	99.9	100.0
6	Wealthiest	vrota_full	95.8	94.3	96.9

Rota_full is 2 or 3 doses of rotavirus vaccine, depending on the national schedule.

* Wilson binomial confidence intervals were calculated for proportion of zero and 100.

Table S6 - Proportions of vaccine coverage based upon caregiver's recall.

Vaccine	Contribution of recall (%)
BCG	21.7
POLIO1	19.9
POLIO3	14.6
DPT1	20.5
DPT3	10.2
MCV	22.5
PCV1	19.5
PCV3	14.6
ROTA1	19.4
ROTA_FULL	14.6
